# Enhancing the Functionalities of Personal Health Record Systems: Empirical Study Based on the HL7 Personal Health Record System Functional Model Release 1

**DOI:** 10.2196/56735

**Published:** 2024-10-09

**Authors:** Teng Cao, Zhi Chen, Masaharu Nakayama

**Affiliations:** 1 Department of Medical Informatics, Tohoku University Graduate School of Medicine, 2-1 Seiryo-machi, Aoba-ku, Sendai, Miyagi, 980-8574, Japan, 81 227177572, 81 227178022

**Keywords:** fast healthcare interoperability resources, logical observation identifiers names and codes, personal health record system functional model, personal health records

## Abstract

**Background:**

The increasing demand for personal health record (PHR) systems is driven by individuals’ desire to actively manage their health care. However, the limited functionality of current PHR systems has affected users’ willingness to adopt them, leading to lower-than-expected usage rates. The HL7 (Health Level Seven) PHR System Functional Model (PHR-S FM) was proposed to address this issue, outlining all possible functionalities in PHR systems. Although the PHR-S FM provides a comprehensive theoretical framework, its practical effectiveness and applicability have not been fully explored.

**Objective:**

This study aimed to design and develop a tethered PHR prototype in accordance with the guidelines of the PHR-S FM. It sought to explore the feasibility of applying the PHR-S FM in PHR systems by comparing the prototype with the results of previous research.

**Methods:**

The PHR-S FM profile was defined to meet broad clinical data management requirements based on previous research. We designed and developed a PHR prototype as a web application using the Fast Healthcare Interoperability Resources R4 (FHIR) and Logical Observation Identifiers Names and Codes (LOINC) coding system for interoperability and data consistency. We validated the prototype using the Synthea dataset, which provided realistic synthetic medical records. In addition, we compared the results produced by the prototype with those of previous studies to evaluate the feasibility and implementation of the PHR-S FM framework.

**Results:**

The PHR prototype was developed based on the PHR-S FM profile. We verified its functionality by demonstrating its ability to synchronize data with the FHIR server, effectively managing and displaying various health data types. Validation using the Synthea dataset confirmed the prototype’s accuracy, achieving 100% coverage across 1157 data items. A comparison with the findings of previous studies indicated the feasibility of implementing the PHR-S FM and highlighted areas for future research and improvements.

**Conclusions:**

The results of this study offer valuable insights into the potential for practical application and broad adoption of the PHR-S FM in real-world health care settings.

## Introduction

Personal health records (PHRs) are beneficial tools in modern health care, as they allow patients to access clinical information and share it with medical staff in a secure and confidential environment [1]. An increasing number of countries, including the United States [2,3], Japan [4,5], European Union-member nations [6,7], South Korea [8,9], and Indonesia [10], have invested significantly in the development and promotion of PHR systems to enhance the health care experience by improving patients’ ability to manage their health information. However, despite the acknowledged benefits of PHRs and substantial investments by various countries, the usage rates of PHR systems have fallen short of expectations [11-13]. In the United Kingdom, over 48.7% of individuals have never interacted with any web-based patient-information-management service [14]. Similarly, in the United States, data from the 2023 Health Information National Trends Survey revealed that more than 43% of people had not accessed their web-based medical records or patient portals even once in the past year [15].

The Personal Health Record System Functional Model Release 1 (PHR-S FM) [16] was developed as a standardized framework for managing personal health information and has been certified as ISO/HL7 (International Organization for Standardization/Health Level Seven International) 16527 [17]. This model outlines a range of possible functionalities in PHR systems, aiming to provide a standardized framework for designing, developing, and evaluating them. The PHR-S FM includes three main sections: (1) personal health (PH), which manages individual health data, encompassing a wide range of functionalities, from medical history to ongoing health conditions; (2) supportive (S), which facilitates the administrative and financial aspects of health care, thereby enabling smoother patient-provider interactions and backend processes; and (3) information infrastructure (IN), which ensures information privacy, security, interoperability, and ease of use. These 3 main sections contain several subsections, each with various functions. The sections and subsections delineate broad functional domains, whereas the functions offer detailed specifications of the features required in PHRs, adhering to a defined parent-child relationship. Each function is characterized by a function ID and Name and is described by a Statement, with its numbering indicating the parent-child relationship between sections and subsections. For example, a function ID “PH.3.1” would be the parent of “PH.3.1.1.”

The PHR-S FM allows researchers to select appropriate functionalities to create a functional profile, which defines a subset of functionalities, thereby facilitating the implementation of PHR. Although the PHR-S FM provides a comprehensive theoretical framework, its practical effectiveness and applicability have not been fully explored. Researchers must precisely specify the actionable functions when creating profiles. However, due to the potential limitations of individual cases, variations may exist in the chosen function list, even if the research objectives are the same.

Harahap et al [18] conducted an extensive systematic review to identify the fundamental functionalities and challenges of the current PHRs. They thoroughly analyzed the essential functionalities required for effective and user-centric PHR systems and created a comprehensive PHR-S FM functional profile containing a subset of functions. This profile encompasses health and administrative records, medication management, communication, appointment management, education, and self-health monitoring. It also considers the challenges faced in PHR implementation, such as interoperability, security and privacy, usability, and data quality. Their functional profile provides a holistic framework for designing and developing PHR systems, ensuring that the systems meet health care users’ evolving needs, thereby enhancing the effective deployment and user adaptation of PHR systems.

Building on previous research, this study aimed to design and develop a tethered PHR system prototype to determine the feasibility of providing servers that meet the PHR-S FM function profile. Moreover, this study examined the efficacy of the PHR-S FM by comparing its contents to that of previous studies.

## Methods

### Personal Health Record System Functional Model

We conducted a comprehensive analysis of Harahap’s PHR-S FM profile and dissected each function into its subfunctions to create actionable levels. For instance, we expanded PH.2.5 into PH.2.5.1–PH.2.5.11, PH.3.1 into PH.3.1.1 and PH.3.1.2, and IN.2 into IN.2.1–IN.2.3.

Based on this classification, we created a new PHR-S FM profile to meet the broad requirements of clinical data management and ensure practical feasibility. We prioritized and implemented functions within the PH module that manage widely used core clinical data [7,19], such as medication lists (PH.2.5.6) and test results (PH.2.5.3). We omitted certain functions, including surgical history (PH.2.5.7), family health history (PH.2.5.8), genetic information (PH.2.5.9), social history (PH.2.5.10), nutrition and diet information (PH.2.5.11), communication with home monitoring devices (PH.3.1.2), medication management (PH.3.4), health education (PH.4), and appointment scheduling (PH.6.3). Although these functions are theoretically important, their operational complexity and the substantial resources required for their analysis exceeded the practical scope of this study.

Furthermore, we integrated essential auxiliary functions from the IN and S modules to support the effective operation of the PH module. These included displaying health records, ensuring system interoperability, and managing user access control. However, we excluded functions such as present ad hoc views of the health record (IN.1.3), standards version control (IN.2.2), application integration (IN.2.3), interoperability protocols (IN.2.4), secure messaging (IN.3.10), and insurance management (S.2.1), as they provided limited support and could unnecessarily complicate the prototype.

Finally, we established the PHR-S FM profile for this study (Table 1). In addition, we extracted detailed descriptions of each function from the ISO/HL7 16527 [17] standard (see Multimedia Appendix 1 for more details).

**Table 1.** The Personal Health Record System Functional Model Release 1 profile function list.

Function list sections	ID #
PH^a^	PH.1.1, PH.1.2, PH.2.5.1, PH.2.5.2, PH2.5.3, PH2.5.4, PH2.5.5, PH2.5.6, PH3.1.1
S^b^	S.1.3, S.1.5
IN^c^	IN.2.1, IN.3.3, IN.4

a PH: Personal Health

b S: Supportive

c IN: Information Infrastructure

### PHR Design and Development

Based on the PHR-S FM profile from this study, we designed the functionality of a PHR prototype. It encompasses 8 distinct functions in the PH section: user demographics (PH.1.2), diagnosis information (PH.2.5.1), medications (PH.2.5.2), imaging test reports and laboratory test reports (PH.2.5.3), allergy information (PH.2.5.4), immunization (PH.2.5.5), visiting records (PH.2.5.6), and vital signs and Patient-Generated Health Data (PGHD; PH.3.1.1) [20]. IN.2.1 explicitly emphasizes the importance of interoperability standards in supporting information sharing between PHR and other systems. We adopted the Fast Healthcare Interoperability Resources Release 4 (FHIR R4) standard [21] to facilitate effective data exchange. FHIR has been widely recognized to effectively overcome data-sharing difficulties between medical information systems and has already become the preferred standard for achieving interoperability [22-24]. Table 2 lists the PHR data categories and their corresponding FHIR R4 resources. This illustrates the 8 types of medical data incorporated into the PHR prototype, specific details captured for each data type, and corresponding FHIR R4 resources. This mapping is essential to ensure data consistency and facilitate interoperability. Moreover, to ensure data consistency and achieve semantic interoperability, we standardized the user-entered PGHD data using the Logical Observation Identifiers Names and Codes (LOINC) system [25] and Unified Code of Units of Measurement (UCUM) system [26] and stored it in the FHIR Observation resources (see Multimedia Appendix 2 for details).

**Figure 1.**
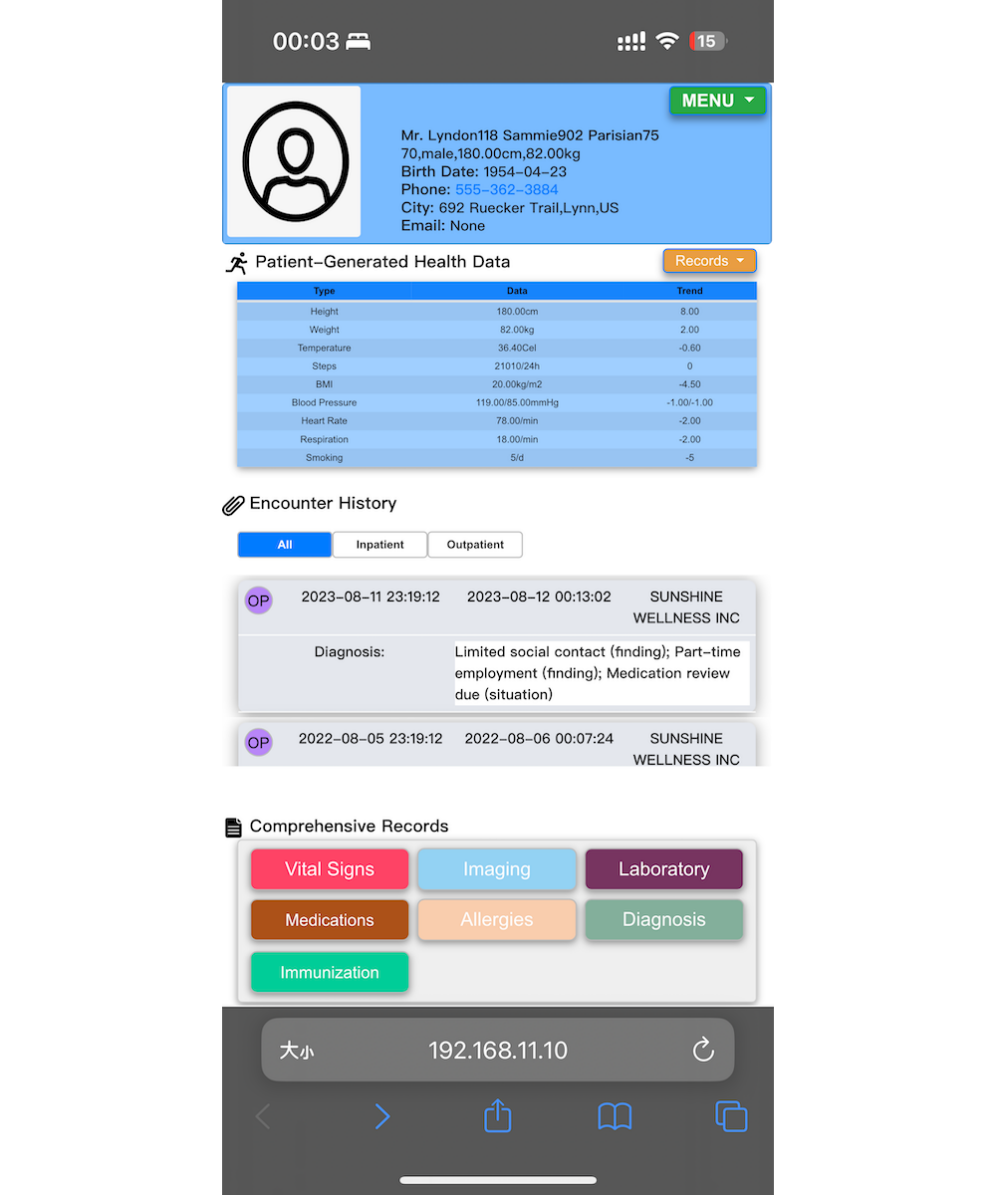
The main interface of the personal health record (PHR) prototype.

This study used Firely R4 [27] as the FHIR server and successfully developed a PHR prototype based on the PHR-S FM. The PHR prototype was designed as a web application optimized for mobile phones. When users log in, the PHR prototype actively retrieves their medical records to ensure that they always have the latest data. Figure 1 shows the main interface with a clear layout that allows users to access essential functions quickly and intuitively. The prototype is divided into 4 main sections: User Demographics, PGHD, Encounter History, and Comprehensive Records (see Multimedia Appendix 3 for details on the implementation of the PHR prototype, functional demonstration, and verification of consistency with the PHR-S FM functional profile).

**Table 2.** Mapping table from PHR^a^ prototype functions to FHIR^b^ resources.

PHR-S FM ID#	PHR functions	Details		FHIR resources
PH.1.1, PH.1.2	User Demographics	Name, Gender, Birthday, Age, Phone Number, Address, Email.		Patient
PH.2.5.1	Diagnosis Information	Disease, Date, Doctor.		Condition, Practitioner
PH.2.5.2	Medications	Item Name, Dose/Unit, Way, Frequency, Start-time, End-time, Amount, Group, Property, Doctor.		Medication Request, Practitioner
PH.2.5.3	Test Results	Imaging Test reports: Item Name, Operate Time, PDF Report, Imaging Test Report, Critical Value.conclusion, Conclusion, Performer.		Diagnostic Report, Observation, ImagingStudy, Practitioner
PH.2.5.3	Test Results	Laboratory Test reports: Item Name, Operate Time, PDF Report, Item Detail, Value, Normal Range, Critical Value/ Detailed Test Names, Critical Value / value, Critical Value/ ReferenceRange, Critical Value / abnormal Flag, Performer.		DiagnosticReport, Observation, Practitioner
PH.2.5.4	Allergy Information	Allergen, Note Date, Severity, Reaction, Comment, Performer.		AllergyIntolerance, Practitioner
PH.2.5.5	Immunization	Vaccine Name, Datetime.		Immunization
PH.2.5.6	Visiting Records	Admission Time, Discharge Time, Visit Department, Hospital, Visit Type, Diagnosis, Doctor.		Encounter, Condition, Practitioner, Organization
PH.3.1.1	Observations and Care	Vital Signs: Datetime, Height, Weight, Temperature, Pulse Rate, Heart Rate, BMI Respiratory Rate, Blood Pressure, Pain Severity, Pediatric Head Occipital-Frontal Circumference Percentile, Body Mass Index (BMI) for Age, Pediatric Weight for Height, Head Occipital-Frontal Circumference, Doctor.		Observation, Practitioner, Organization
PH.3.1.1	Observations and Care	Patient-Generated Health Data (PGHD): Height, Weight, Temperature, Steps, BMI, Blood Pressure, Heart Rate, Respiration Rate, Smoking Habits.		Observation, Organization

a PHR: personal health record.

b FHIR: Fast Healthcare Interoperability Resources R4.

### Validation

We used the Synthea dataset [28] to validate the effectiveness of the PHR prototype. Synthea was designed to generate realistic and synthetic medical records and has been extensively applied in multiple studies [29-32]. It extracts data from public datasets and statistical models, ensuring their authenticity and representativeness without compromising individual privacy. The dataset comprised comprehensive health records for over 1 million fictional patients in standardized formats, such as HL7 FHIR, C-CDA, and CSV. These records encompassed a wide range of medical information, including medication usage, allergies, medical history, and social health determinants, making them extremely valuable for developing, testing, and demonstrating PHR systems.

We randomly selected 5 patients from the Synthea dataset to validate the effectiveness of the PHR prototype. Their medical data were formatted in FHIR R4 and uploaded to the Firely server for testing. We analyzed FHIR resources and counted the number of FHIR resource types for each patient. The results were then compared with the types and quantities of data displayed in the PHR prototype. The evaluation method involved a quantitative analysis of the number of data entries available in the Synthea dataset against the number displayed in the PHR system, aiming to ascertain the prototype’s effectiveness in data representation.

### Ethical Considerations

As the Synthea dataset comprised solely synthetic data unrelated to any real individuals, considerations of legal and privacy issues were not required. Moreover, these data could be used for research without requiring patient consent or institutional review board approval.

### Comparison With Prior Research

In our study, we conducted a comprehensive search of databases such as PubMed, IEEE Xplore, and ACM Digital Library using the keywords “PHR,” “PHR-S FM,” and “Personal Health Record System Functional Model.” The search yielded 4 studies [33-36], but only 3 of these detailed the PHR-S FM functional profiles used as well as the implementation of PHR functionalities. Specifically, Katehakis et al [33] mentioned a PHR system for chronic care and home health, using the PHR-S FM and experiences from EU projects to ensure interoperability and meet health management needs. The project underscored the importance of adhering to standards like the HL7 PHR-S FM for better adoption of PHR systems. Saripalle et al [34] used HL7 FHIR to design an interoperable mobile PHR following PHR-S FM guidelines, achieving integration with OpenEMR and underscoring the benefits of a modular approach and standards-based API-driven data exchange. Chatterjee et al [35] developed a tethered PHR using FHIR resources and SNOMED-CT (Systematized Nomenclature of Medicine–Clinical Terms) terminology according to PHR-S FM guidelines, focusing on collecting PGHD from diverse sources for electronic health record integration.

We analyzed and quantified the PHR-S FM functions in these 3 studies [33-35]. Using the PHR-S FM functional profile proposed by Harahap et al [18] as a benchmark, we conducted a comparative analysis of the results of these studies and of the present study to quantify the implementation of each functionality outlined in the profile. We conducted a survey on how many studies had implemented each function. The results were then subjected to a detailed discussion to ascertain the strengths and weaknesses of each PHR-S FM function. This approach aimed to evaluate the feasibility of the PHR-S FM framework and identify potential directions for future research.

## Results

### PHR Prototype Functionality

**Figure 2.**
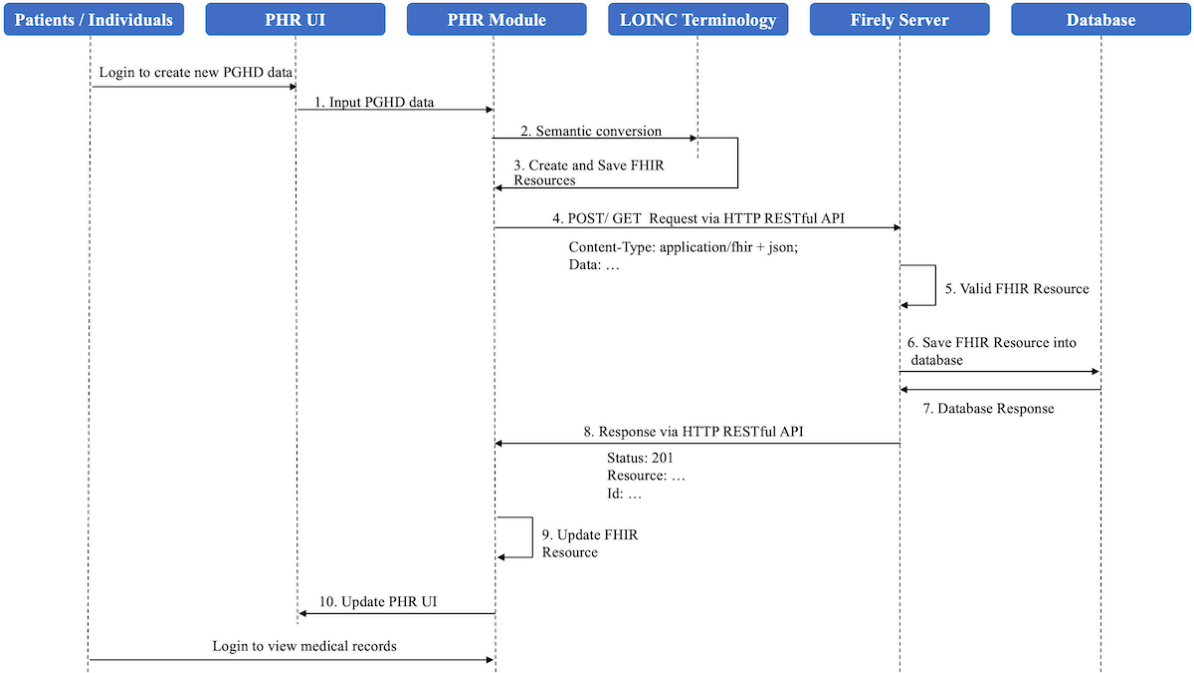
Sequence diagram of the synchronization of data between PHR and FHIR server.

In this section, we verify and demonstrate the functionality of the PHR prototype (see Multimedia Appendix 4 for details). Figure 2 shows the sequence of events for communicating the FHIR resources between the PHR and FHIR servers. It outlines all the interactions between the PHR prototype and the Firely server based on FHIR resources. The PHR prototype enables users to create new PGHD, which undergo semantic conversion to ensure consistent terminology and units using LOINC and UCUM standards, then synchronizing them to the FHIR server as FHIR Observation resources. Concurrently, the PHR prototype demonstrates interoperability with the Firely server by using the RESTful API, efficiently retrieves various medical data, including allergies, immunization, vital signs, medications, diagnostic information, and laboratory and imaging test reports, while also uploading newly generated PGHD data back to the Firely server.

### Validation

We conducted a quantitative analysis of the PHR prototype using data from 5 patients extracted from the Synthea dataset (See Multimedia Appendix 5 for an example of verifying that the PHR correctly displayed data from Synthea). We identified 1157 data items across these patient profiles, with individual counts of 183, 154, 198, 327, and 295 (see Multimedia Appendix 6). The prototype accurately displayed all data entries contained within the patient profiles, achieving a coverage rate of 100%.

Entries were marked as not applicable if corresponding data points were absent in the patient profiles. The PHR prototype was designed to display 21 distinct FHIR resources and effectively presented data entries covering 20 out of 21 health data categories (95%), including user demographics, diagnosis information, medications, and laboratory test reports.

Specifically, for imaging test reports, no entries were recorded for any of the 5 patients. The PHR prototype could process and display such data, mirroring its performance with allergy information. Despite the absence of data in 4 out of the 5 patient profiles in the allergy category, the prototype successfully displayed all 7 entries for the 1 profile that included allergy data. This demonstrated the prototype’s capability to fully render allergy information available in the dataset. This suggested that imaging test reports would be similarly displayed with complete accuracy if these data were available.

### Comparison With the Findings of Previous Studies

This study used the PHR-S FM functional profile, as proposed by Harahap et al [18], as a foundational benchmark for conducting a comprehensive comparative analysis of 3 studies that used it. Table 3 presents a comparative analysis of our study with 3 other studies based on the PHR-S FM framework. In Table 3, “✓” was used to identify implemented functions, and “-” was used for unspecified functions. The results indicated that 5 functions were adopted and implemented in 4 studies, all of which originated from the PH module. Another 5 functions were implemented by 3 studies (3/4), 4 of which belonged to the PH module, and 1 was an interoperability function of the IN module. Furthermore, 2 functions were implemented in half of the studies (2/4), including the PH.2.5.10 (Manage Social History) function of the IN module. Moreover, 4 functions were implemented in 1 study (1/4). Of these, the “Manage Surgical History” function attracted attention, as it was the only function of the PH module implemented by only 1 study. In total, 13 functions were not implemented in any study.

**Table 3.** Comparative analysis of specific function completions in the PHR-S FM profile.

Function list sectionsID #		Function name	Saripalle et al [34]	Chatterjee et al [35]	Katehakis et al [32]	This study	Function score
**PersonalHealth (PH)**							
	PH.1.1	Identify and Maintain a PHR Account Holder Record	—	✓	✓	✓	3
	PH.1.2	Manage PHR Account Holder Demographics	✓	✓	✓	✓	4
	PH.2.5.1	Manage Problem Lists	✓	✓	✓	✓	4
	PH.2.5.2	Manage Medication List	✓	—	✓	✓	3
	PH.2.5.3	Manage Test Results	✓	✓	✓	✓	4
	PH.2.5.4	Manage Allergy, Intolerance, and Adverse Reaction List	✓	✓	✓	✓	4
	PH.2.5.5	Manage Immunization List	✓	—	✓	✓	3
	PH.2.5.6	Manage Medical History	✓	✓	—	✓	3
	PH.2.5.7	Manage Surgical History	—	—	✓	—	1
	PH.2.5.8	Maintain Family History	—	—	—	—	0
	PH.2.5.9	Manage Personal Genetic Information	—	—	—	—	0
	PH.2.5.10	Manage Social History	✓	✓	—	—	2
	PH.2.5.11	Nutrition and Diet Information	—	—	—	—	0
	PH.3.1.1	Manage Personal Observations and Care	✓	✓	✓	✓	4
	PH.3.1.2	Communication with Home Monitoring Devices	—	—	—	—	0
	PH.3.4	Manage Medications	—	—	—	—	0
	PH.4	Manage Health Education	—	—	—	—	0
	PH.6.3	Communications between Provider and/or the PHR Account Holder’s Representative	—	—	—	—	0
**Supportive (S)**							
	S.1.3	Manage HealthCare Provider Information	—	—	—	✓	1
	S.1.5	Manage Healthcare Facility Information	—	—	—	✓	1
	S.2.1	Capture and Read Health Insurance Account and Benefit Information	—	—	—	—	0
**Information Infrastructure (IN)**							
	IN.1.3	Present Ad Hoc Views of the Health Record	—	—	—	—	0
	IN.2.1	Interoperability Standards	✓	✓	-	✓	3
	IN.2.2	Interoperability Standards Versioning and Maintenance	—	—	—	—	0
	IN.2.3	Standards-Based Application Integration	—	—	—	—	0
	IN.2.4	Interoperability Agreements	—	—	—	—	0
	IN.3.3	Entity Access Control	—	✓	—	✓	2
	IN.3.10	Secure Messaging	—	—	—	—	0
	IN.4	Auditable Records	—	—	—	✓	1

## Discussion

### Principal Results

This study developed a PHR prototype guided by the PHR-S FM profile, which successfully managed medical data, including immunizations, allergies, vital signs, medications, diagnoses, and test results. The prototype used LOINC and FHIR R4 for semantic consistency and standardization of user-input data, ensuring data consistency and interoperability. The prototype was validated using the Synthea dataset and demonstrated 100% coverage in accurately displaying patient information. A comprehensive analysis of previous studies revealed the current implementation status of the PHR-S FM framework, which predominantly focuses on the PH module with limited emphasis on the IN and S modules. To the best of our knowledge, this was the first study to examine the feasibility of using PHR-S FM to develop and design PHRs.

### Comparison With the Findings of Previous Studies

#### PHR-S FM Feasibility Analysis

The detailed analysis of the functional implementation revealed significant trends and differences (Table 3). The diversity in the extent of functionality implementation indicated varying levels of focus on specific features in the PHR-S FM framework across different studies.

##### Widely Implemented Functions

As shown in Table 3, 10 features, including PH.1.2 (Manage PHR Account Holder Demographics), PH.2.5.1 (Manage Problem Lists), and PH.2.5.3 (Manage Test Results), were implemented by at least 3 studies, of which 9 were attributed to PH modules. We found that almost all these functions aligned closely with the PHR core set of necessary criteria outlined in the ONC’s Meaningful Use criteria [7]. The widespread adoption of these features may be related to their centrality in patient data management and essential compliance with healthcare standards [7,19].

IN.2.1 (Interoperability Standards), the only function not corresponding to the PH module, is a major theme in current PHR research; however, with the advent of FHIR, interoperability can be achieved [37]. FHIR released the first version with normative content (FHIR R4) in 2019. Katehakis et al [33] denoted a missed opportunity for interoperability. This indicated that researchers should focus on keeping up to date with the latest technologies related to PHRs to expand and enhance their functionality. In addition, the PHR-S FM community should undertake more initiatives to promote FHIR adoption and improve the interoperability and use of PHR systems. In comparison to the integrated PHR by Katehakis et al [33], both Saripalle et al and Chatterjee et al [35] developed tethered PHRs using FHIR for interoperability. While Saripalle et al [34] integrated multiple health information, it lacked qualitative and quantitative evaluations. Chatterjee et al [35] focused on recording PGHDs, but whether the prototype could effectively retrieve and display medical data from institutions was unclear.

##### Limited Implementation Functions

Six features were implemented in at least one study. One function, PH.2.5.10 (Manage Social History), was partially implemented in this study, allowing smoking status to be recorded; however, it was not fully implemented due to the variety of data types involved and the complexity of data collection [38].

The PH.2.5.7 (Manage Surgical History) function was uniquely mentioned in Katehakis et al’s study [33], which effectively applied the PHR-S FM framework in the context of real research projects in the European Union. This allowed researchers to bypass the complexities of implementing this detailed function. In contrast, the other 3 studies were at the prototype stage. However, other functions mentioned by Katehakis et al [33] were adopted by at least 2 other studies, highlighting the feasibility of the PHR-S FM framework across various research settings.

##### Functions Not Mentioned

Many functions were not mentioned because the implementation of certain features was not within the scope or the priority of a particular study. Moreover, collecting data for the PHR system was challenging, and some collected data were not useful. For instance, the intricacy of PH.2.5.9 (Manage Personal Genetic Information) and PH.2.5.11 (Nutrition and Diet Information) were infrequently used in PHRs [39]. The limited participation of the IN and S module functions may be attributed to the complexity of their implementation, such as IN.3.10 (Secure Messaging), which ensures the security of communication [40]. Although this function is significant in the PHR system, it faces technical challenges [11]. The S.2.1 (Capture and Read Health Insurance Account and Benefit Information) function was not implemented, likely due to the need for data from multiple sources and a wide variety of types, which rendered it costly and challenging to implement [41]. PHR conformed to the PHR-S FM functional profile, allowing researchers to effectively use it by tailoring the functional profile to suit their research needs. This flexibility emphasized the adaptability of PHR-S FM and demonstrated its use as a dynamic health care research field, despite challenges in achieving certain features.

Furthermore, this study uncovered a significant trend, with the S and IN modules used less frequently than the PH modules. The S module offers users administrative and financial support functionalities, and its implementation can enhance the effectiveness of health care services. Implementing the IN module ensures privacy, security, and interoperability, facilitating access and usability of PHR functions. Both modules are essential components of a comprehensive PHR system. Therefore, researchers should prioritize the S and IN modules when considering PH module functionalities, as they form the backbone that supports the optimal operation of PHRs.

### Comparison With Studies Based on Other PHR Frameworks

To delve deeper into the development of PHR systems, this study conducted a comparative analysis with research based on the PHR-S FM and examined other significant studies. Lee et al [42], Li [43], and Song et al [44] presented further perspectives on PHR systems’ development. Lee et al [42] developed a PHR prototype that organizes and visualizes personal health information according to a patient-centered journey map. However, their study neither achieved data interoperability nor enabled users to record PGHD data, making the application irrelevant for people without diseases. Li [43] proposed a service-oriented approach to integrating the electronic health record and PHR systems. However, the development and implementation of a service-oriented approach-based environment can be highly complex and expensive. Moreover, compared with the FHIR, they used the HL7 CDA to deliver messages, thereby increasing maintenance difficulty. Song et al [44] developed a patient summary application based on the international patient summary and FHIR R4 standards to mitigate information overload and reduce physicians’ and nurses’ workloads. Unlike traditional PHRs, which provide a comprehensive display of numerous information items, this application is limited in scope, displaying only the essential international patient summary standard information on a single screen. This design choice aims to simplify the user interface for clinicians but may restrict the availability of more detailed patient data. Thus, this study implemented more functions of the PHR-S FM and created an advanced PHR with more comprehensive functions that can better meet users’ functional needs than those of previous studies.

### Limitations

Although this study exhaustively explored the feasibility of PHR-S FM functionality, its scope was limited to 29 functions. As the PHR-S FM is a comprehensive framework designed to encompass the full range of functions that may be integrated into a PHR system, this study did not address its full potential. Future research could consider a broader set of features to more thoroughly evaluate the value of the PHR-S FM for practical applications.

In addition, the application developed in this study was a prototype and must be verified for use and evaluated for functionality in the future. Furthermore, the proposed prototype does not currently address privacy and security concerns. The platform SMART on FHIR [45] offers secure access control through the OAuth2 standard, ensuring that only authorized users and applications can access a patient’s PHR. Future research should delve deeper into the integration of this technology in PHRs. Finally, the data types included in the PHR in this study were limited and lacked information on appointments and surgical history. However, our modularly designed PHR allows for the addition of such content in the future without altering the foundational structure.

### Conclusions

This study developed a PHR prototype based on the PHR-S FM profile and compared its results with the findings of previous studies to evaluate the framework’s feasibility in the application of PHR. The findings demonstrated the us of the PHR-S FM as a valuable tool for PHR development and highlighted its potential to inform future technological enhancements in the PHR domain. The empirical data on function implementation offer a foundation for subsequent PHR system design. Moreover, this study provides actionable recommendations for applying the PHR-S FM in health care settings. The adaptability of the PHR-S FM framework is well suited to evolving health information management demands, suggesting avenues for continued research and optimization.

## Supplementary material

10.2196/56735 Multimedia Appendix 1 The PHR-S FM (Personal Health Record System Functional Model) functional profile details.

10.2196/56735 Multimedia Appendix 2 Mapping table between the PGHD (Patient-Generated Health Data) and the LOINC (Logical Observation Identifiers Names and Codes) code systems.

10.2196/56735 Multimedia Appendix 3 The development of the personal health record application.

10.2196/56735 Multimedia Appendix 4 The details of the personal health record application.

10.2196/56735 Multimedia Appendix 5 Verification of personal health record display for Synthea immunization data.

10.2196/56735 Multimedia Appendix 6 Comprehensive data display coverage of the personal health record prototype Across 5 patient profiles.

## Abbreviations

FHIR: Fast Healthcare Interoperability Resources
FM: Personal Health Record System Functional Model
HL7: Health Level Seven
HTML5: HyperText Markup Language 5
IN: information infrastructure
ISO/HL7: International Organization for Standardization/Health Level Seven International
LOINC: Logical Observation Identifiers Names and Codes
PGHD: Patient-Generated Health Data
PH: personal health
PHR: personal health record
S: supportive
SNOMED-CT: Systematized Nomenclature of Medicine-Clinical Terms
UCUM: Unified Code of Units of Measurement
