# Supplementary material for: Enhancing the Functionalities of Personal Health Record Systems: Empirical Study Based on the HL7 Personal Health Record System Functional Model Release 1
Source: JMIR Med Inform. 2024 Oct 9;12:e56735. doi: 10.2196/56735 (PMC11481820; doi:10.2196/56735)
Supplement: Multimedia Appendix 1 [file medinform-v12-e56735-s001.pdf]

Multimedia Appendix 1

Table S1. The PHR-S FM functional profile details [17].

Function List Sections	ID #	Name	Statement
**Personal Health (PH)**	**PH.1.1**	Identify and Maintain a PHR Account Holder Record	Unambiguously identify the PHR Account Holder; correctly link the information with the PHR Account Holder and vice-versa.
	**PH.1.2**	Manage PHR Account Holder Demographics	Enable the PHR Account Holder to manage information about demographics.
	**PH.2.5.1**	Manage Problem Lists	Manage the PHR Account Holder's health problem list and provide the ability to manage the problem list over time in accordance with organizational policy and/or jurisdictional law.
	**PH.2.5.2**	Manage Medication Lists	Manage the PHR Account Holder's medication list.
	**PH.2.5.3**	Manage Test Results	Manage the results of diagnostic tests including inpatient, ambulatory, and home-monitoring tests.
	**PH.2.5.4**	Manage Allergy, Intolerance, and Adverse Reaction List	Manage the PHR Account Holder's list of known allergens and adverse reactions with all pertinent information.
	**PH.2.5.5**	Manage Immunization Lists	Manage the PHR Account Holder's immunization data and associated capabilities, including reminders, alerts, compliance, and administration.
	**PH.2.5.6**	Manage Medical History	Manage the PHR Account Holder's medical history.
	**PH.2.5.7**	Manage Surgical History	Manage the PHR Account Holder's history of surgical procedures.
	**PH.2.5.8**	Maintain Family History	Manage the PHR Account Holder's family health history.
	**PH.2.5.9**	Manage Personal Genetic Information	Manage the PHR Account Holder's genetic information.
	**PH.2.5.10**	Manage Social History	Manage the PHR Account Holder's social history, including health-related habits and risk factors.
	**PH.2.5.11**	Nutrition and Diet Information	Manage the PHR Account Holder's nutrition and diet-related information.

	**PH.3.1.1**	Manage Personal Observations and Care	Enable the PHR Account Holder's ability to access personally sourced data and make it available electronically to authorized healthcare provider(s) and other authorized users and applications.
	**PH.3.1.2**	Communication with Home Monitoring Devices	Enable the PHR Account Holder to capture and view home-monitoring device data and electronically make it available to authorized healthcare provider(s) and other authorized users and applications.
	**PH.3.4**	Manage Medications	Assist the PHR Account Holder in managing their individual medications.
	**PH.4**	Manage Health Education	Provide reliable patient education and information customized to the patient, based on the information in the PHR to help the PHR Account Holder explore treatment options.
	**PH.6.3**	Communications Between Provider and/or the PHR Account Holder's Representative	Enable the PHR Account Holder to capture information in preparation for an encounter with a healthcare provider and support ongoing interactions with that provider. The system should enable the PHR Account Holder to request appointments with healthcare providers and capture information in preparation for the encounter.
**Supportive (S)**	**S.1.3**	Manage Healthcare Provider Information	Support the import and retrieval of data necessary to identify a healthcare provider.
	**S.1.5**	Manage Healthcare Facility Information	Support the import and retrieval of data necessary to identify a healthcare facility.
	**S.2.1**	Capture and Read Health Insurance Account and Benefit Information	Enable the PHR Account Holder to request and/or receive and read information on their general health insurance benefits.
**Information Infrastructure (IN)**	**IN.1.3**	Present Ad Hoc Views of the Health Record	Present ad hoc views of the PHR information in accordance with user roles, organizational policies, and jurisdictional laws pertinent to privacy and confidentiality.
	**IN.2.1**	Interoperability Standards	Support the ability to operate seamlessly with other systems, either internal or external, that adhere to recognized interoperability, security, and privacy standards. "Other systems" include other PHR and

			electronic health record systems, applications within a PHR-S, and other authorized entities that interact with a PHR-S.
	**IN.2.2**	Interoperability Standards Versioning and Maintenance	Enable version control according to local policies to ensure the maintenance of utilized interoperability standards.
	**IN.2.3**	Standards-Based Application Integration	Enable application integration based on standards.
	**IN.2.4**	Interoperability Agreements	Support interactions with entity directories to determine the address, profile, and data exchange requirements of known and/or potential partners. Use the rules of interaction specified in the partner's interoperability agreement, including privacy and security requirements, when exchanging information.
	**IN.3.3**	Entity Access Control	Verify and enforce access control to all PHR-S components and PHR information and functions for end-users, applications, sites, and others to prevent unauthorized use of a resource.
	**IN.3.10**	Secure Messaging	Enable secure electronic communication between PHR Account Holders and healthcare providers.
	**IN.4**	Auditable Records	Provide audit capabilities for system access and usage indicating who accessed the record, when, what actions were taken, and when the actions occurred. Examples of auditable actions include creating, modifying, viewing, extracting, and deleting a record.
