# Supplementary material for: Enhancing the Functionalities of Personal Health Record Systems: Empirical Study Based on the HL7 Personal Health Record System Functional Model Release 1
Source: JMIR Med Inform. 2024 Oct 9;12:e56735. doi: 10.2196/56735 (PMC11481820; doi:10.2196/56735)
Supplement: Multimedia Appendix 2 [file medinform-v12-e56735-s002.pdf]

Multimedia Appendix 2

Table S2. Mapping table between the PGHD and the LOINC code systems.

**Data**	**LOINC Code**	**Units**	**FHIR Resource**
Body Height	8302-2	cm	Observation
Body Weight	29463-7	kg	Observation
Body Temperature	8310-5	Cel	Observation
Steps	41950-7	/24h	Observation
Body Mass Index	39156-5	kg/m^2	Observation
Blood Pressure	35094-2	-	Observation
Systolic Blood Pressure	8480-6	mm/Hg	Observation
Diastolic Blood Pressure	8462-4	mm/Hg	Observation
Heart Rate	8867-4	/min	Observation
Respiration Rate	9279-1	/min	Observation
Smoking Habits	63773-6	/d	Observation
