# Supplementary material for: Enhancing the Functionalities of Personal Health Record Systems: Empirical Study Based on the HL7 Personal Health Record System Functional Model Release 1
Source: JMIR Med Inform. 2024 Oct 9;12:e56735. doi: 10.2196/56735 (PMC11481820; doi:10.2196/56735)
Supplement: Multimedia Appendix 3 [file medinform-v12-e56735-s003.pdf]

## Multimedia Appendix 3

## The development of the PHR application

Figure S1 presents a system overview. The system comprises two modules: 1) Firely Server R4, an advanced FHIR server with processors for managing RESTful API interactions and a Facade plugin for data access; and 2) a PHR system engineered for seamless interconnection with the Firely server. Upon user login, the PHR system dynamically fetches pertinent medical data via the RESTful application programming interface (API) requests and renders this information on the user interface. The PHR can also archive PGHD data within the Firely server. This allows users to view the latest medical information each time they log in.

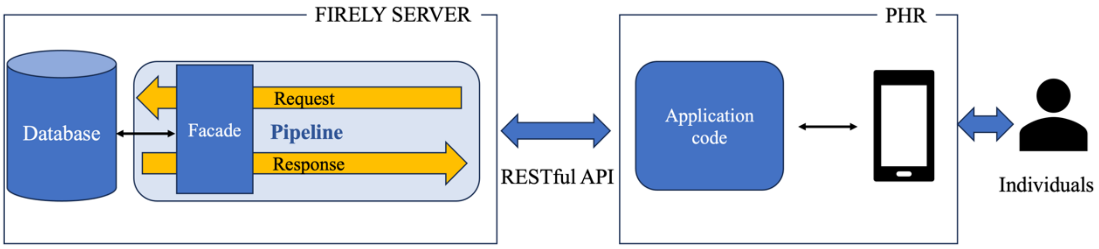


Figure S1. An overview of PHR workflow.

This study engineered a web-based PHR prototype designed for mobile devices to optimize their usability and enable smartphone accessibility. Figure S2 illustrates the core architecture of the web-based PHR prototype. The front-end of the prototype was crafted with HTML5, CSS, and JavaScript; the back-end, built with Python 3.8, includes the Server Interaction and Data Processing components. The Server Interaction component employs the RESTful API for data interaction with the Firely server. The data processing component handles the resources from the FHIR server, passes them to the front-end to update the user interface, and processes the daily data recorded by the front-end as resources for uploading to the server. Two libraries were used in this process: interactions with the FHIR server were conducted using the FHIRClient [1], and web applications were built using Django [2].

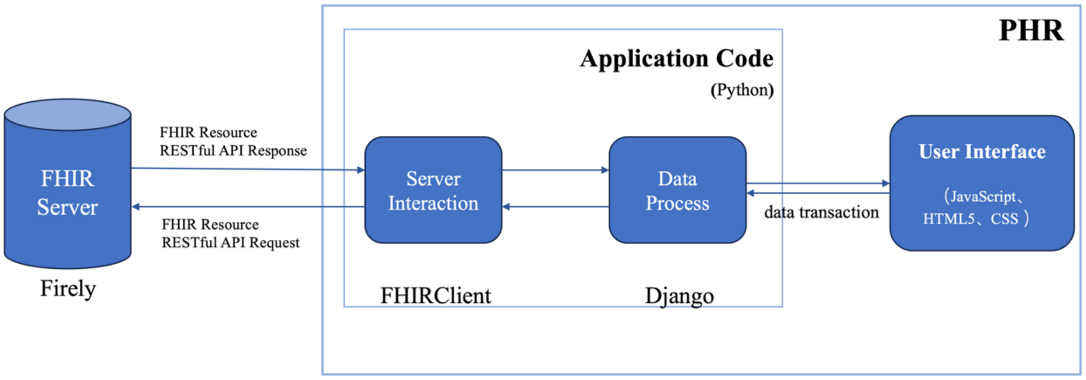


Figure S2. PHR and Firely server interaction flow.

## References


1. FHIR DaH. How to create a FhirClient. https://docs.fire.ly/projects/Firely-NET-SDK/en/stable/client/setup.html [accessed Jun 6, 2024].
2. Foundation DS. Django Project. 2005-2023. https://www.djangoproject.com/ [accessed Jun 6, 2024].
