# Supplementary material for: Enhancing the Functionalities of Personal Health Record Systems: Empirical Study Based on the HL7 Personal Health Record System Functional Model Release 1
Source: JMIR Med Inform. 2024 Oct 9;12:e56735. doi: 10.2196/56735 (PMC11481820; doi:10.2196/56735)
Supplement: Multimedia Appendix 4 [file medinform-v12-e56735-s004.pdf]

Multimedia Appendix 4

Details of the PHR application

User Demographics

This function was designed to manage user demographics, including name, gender, birth date, age, phone number, address, and email (PH.1.2). Each user has a unique username through which the PHR can identify users and correctly link information to them (PH.1.1, IN.3.3). These details can currently be viewed in the PHR; however, modifications are not permitted through the platform.

Patient-Generated Health Data

This function is used to manage PGHD information, including height, weight, temperature, steps, body mass index, blood pressure, heart rate, respiration rate, and smoking habits (PH.3.1.1). On the main interface of the PHR prototype (Figure 1), users can view the latest PGHD records and data trends. Figure S3 shows the user-input interface, which allows users to record nine types of daily data. Newly input PGHD data are semantic, standardized using the LOINC, and stored as FHIR Observation resources (IN.2.1). Figure S4 shows the process of user-input heart rate data integrated into the FHIR Observation resource, employing LOINC semantics (specific code: 8867-4). In addition, the Observation resource's "performer" attribute references the FHIR Organization resource to distinguish the data source (S.1.5).

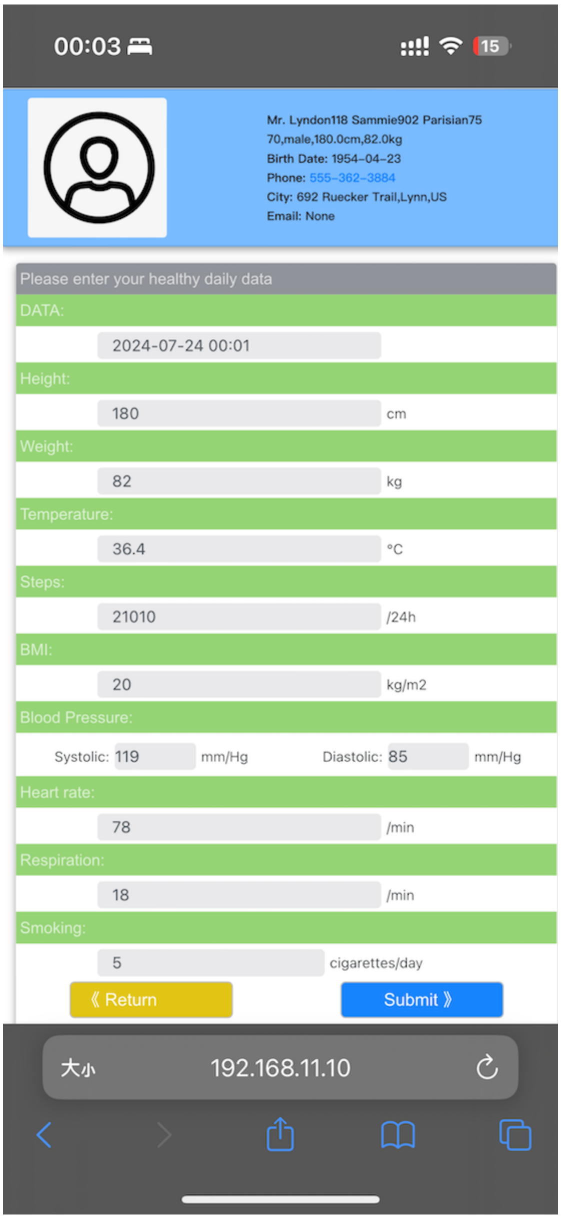

Figure S3. User-input interface of the PHR prototype.

```
{
  "resourceType": "Observation",
  "id": "test-1",
  "status": "final",
  "category": [ … ],
  "code": {
    "coding": [
      {
        "system": "http://loinc.org",
        "code": "8867-4",
        "display": "Heart rate"
      }
    ],
    "text": "Heart rate"
  },
  "subject": {"reference": "https://server.fire.ly/Patient/baede442-d962-45f1-8958-0cb838540ecf"},
  "performer": {"reference": "https://server.fire.ly/Organization/dea39875-4931-43b5-9d3b-6d52142b1cc5"},
  "effectiveDateTime": "2023-01-17T18:32:29Z",
  "valueQuantity": {
    "value": 69,
    "unit": "beats/minute",
    "system": "http://unitsofmeasure.org",
    "code": "/min"
  }
}
```


a. An example of creating "Heart Rate" data by FHIR Observation Resource


```
{
  "resourceType": "Organization",
  "id": "dea39875-4931-43b5-9d3b-6d52142b1cc5",
  "type": [
    {
      "coding": [
        {
          "system": "http://terminology.hl7.org/CodeSystem/organization-type",
          "code": "prov",
          "display": "Healthcare Provider"}]} ],
  "name": "PHR Prototype",
  "telecom": [
    {
      "system": "phone",
      "value": "022-655 2300",
      "use": "work"
    }
  ],
  "contact": [
    {
      "telecom": [
        {
          "system": "phone",
          "value": "080-3338-2334"
        }
      ]
```


b. An example of creating "PHR Prototype" data by FHIR Organization Resource

Figure S4. Example of FHIR observation and organization resources.

Encounter History

This function provides the essential medical records for each encounter, consolidating all relevant data, such as allergies, vital signs, medications, diagnostic information, immunization, and test results, for current encounters into a single, easily navigable location. The PHR defaults to a chronological display of encounter histories, and users can quickly locate their target visit list based on visit type and time (PH.2.5.1). The main interface presents details from the medical institution (S.1.5) and diagnostics from each encounter (Figure 1). FHIR Encounter resources are used to capture detailed information about users' medical encounters, and the "rank" attribute of the Encounter resource is used to distinguish between primary (rank = 1) and secondary diagnoses.

Figure S5 shows the trajectory of blood pressure in vital signs data, reflecting changes during a specific medical encounter (PH.3.1.1). Additionally, the vital signs data includes temperature, pulse, respiratory rate, and so on. Users have the option to select a day, month, or year to view data trends over different periods. Figure S6 shows the allergy interface, which outlines allergy information, including the allergen, noted date, severity, and reaction, for a specific medical encounter (PH.2.5.4). Figure S7 shows the medications interface, which lists medication information, including the medications, dose/unit, mode, frequency, start date, and end date, and labels groups of drugs for a specific medical encounter (PH.2.5.2). Figure S8 shows the laboratory reports interface, which displays trends in laboratory test details over time (PH.2.5.3); users can observe the trends in data changes by selecting specific items. This design allows users to easily access and understand healthcare interactions over time.

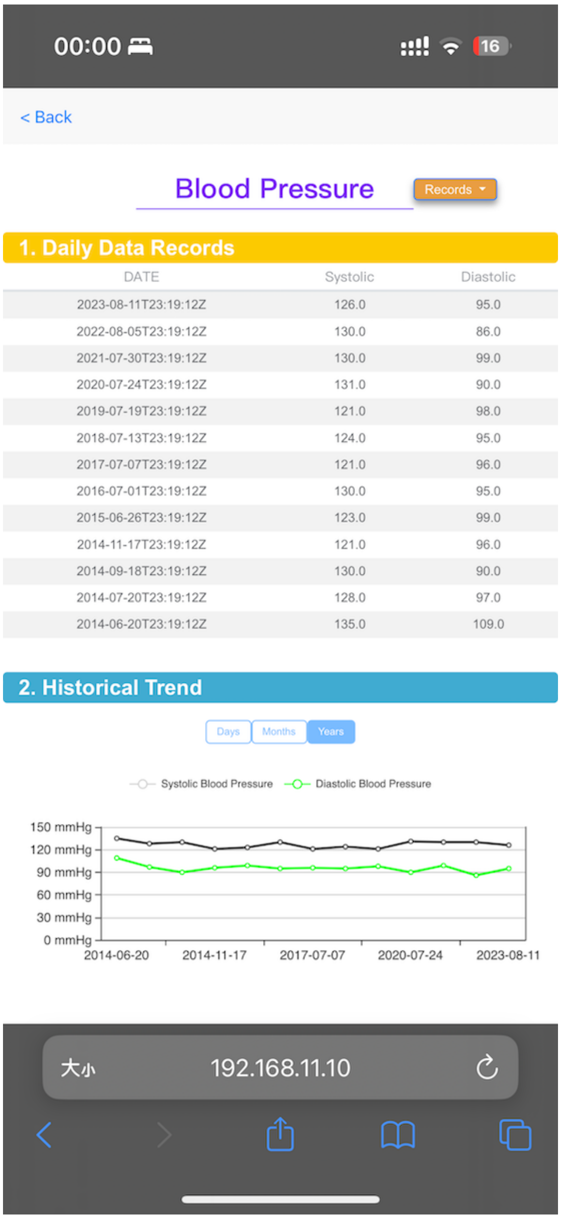

DATE	Systolic	Diastolic
2023-08-11T23:19:12Z	126.0	95.0
2022-08-05T23:19:12Z	130.0	86.0
2021-07-30T23:19:12Z	130.0	99.0
2020-07-24T23:19:12Z	131.0	90.0
2019-07-19T23:19:12Z	121.0	98.0
2018-07-13T23:19:12Z	124.0	95.0
2017-07-07T23:19:12Z	121.0	96.0
2016-07-01T23:19:12Z	130.0	95.0
2015-06-26T23:19:12Z	123.0	99.0
2014-11-17T23:19:12Z	121.0	96.0
2014-09-18T23:19:12Z	130.0	90.0
2014-07-20T23:19:12Z	128.0	97.0
2014-06-20T23:19:12Z	135.0	109.0

Figure S5. Vital signs interface in the “encounter history” section.

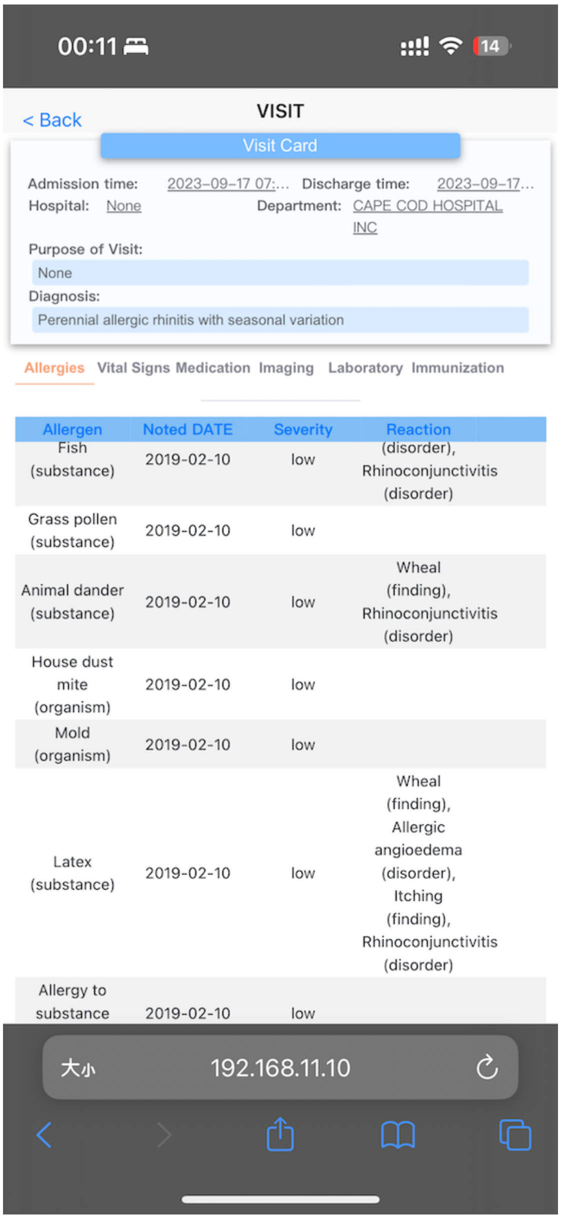

Figure S6. Allergies interface in the "encounter history" section.

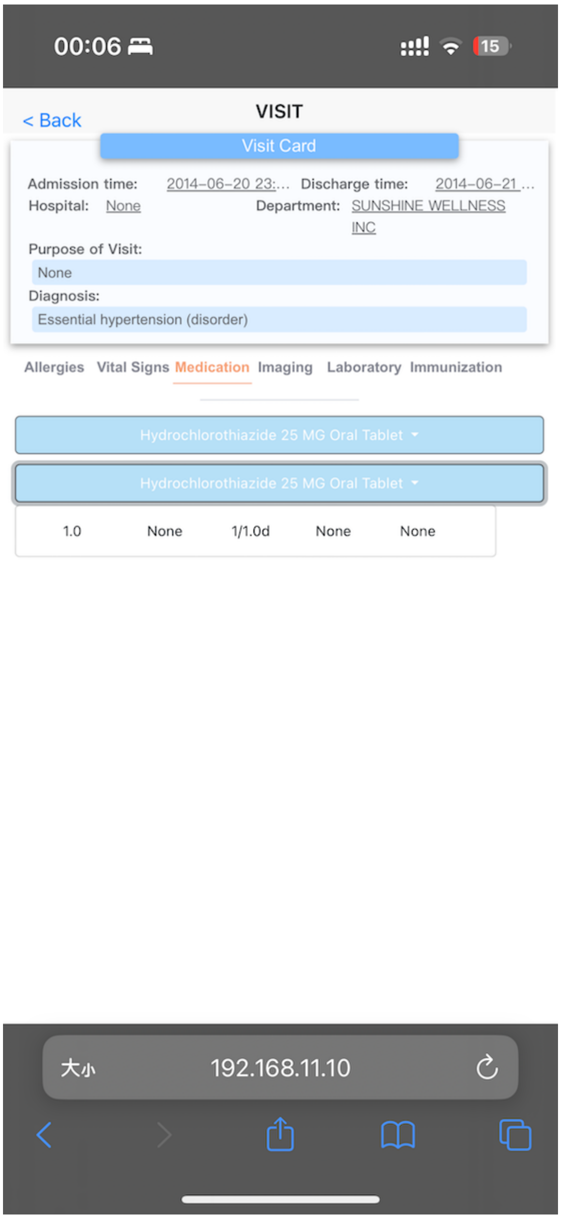

Figure S7. Medications interface in the "encounter history" section.

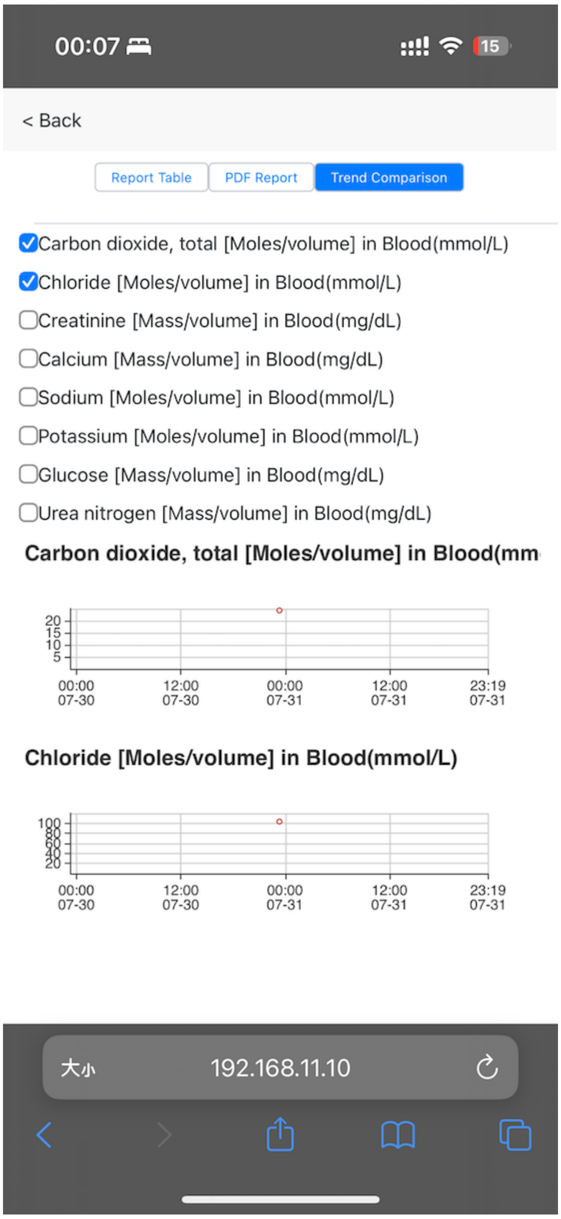

Figure S8. Laboratory reports interface in the “encounter history” section.

Comprehensive Records

The PHR prototype offers two distinct methods of managing medical records, whereas the Comprehensive Records function organizes and summarizes medical records by data type, thereby enabling user-friendly navigation. This allows users to access specific types of medical data directly from the main interface. The Encounter History and Comprehensive Records functions provide users with comprehensive medical history management tools (PH.2.5.6). For instance, Figure S9 shows the diagnosis interface, which delineates the user’s diagnosis history, including diseases, comorbidities, diagnosis dates, and doctors, and provides a clickable link that directly takes the user to the Encounter History interface relevant to the current visit. In the Medical Record function, healthcare provider

information is consistently integrated across all data types, and users can sort the data according to their preferences (S.1.3). Transitioning between the Encounter History and Comprehensive Records functions is rendered effortless through shortcuts. Every medical record entry has a "link" feature that directs users to the corresponding Encounter History section. Figure S10 shows the imaging interface, which presents the user's previous imaging records, including radiology and dates, and provides clickable reports and links that lead to PDF Reports in the Encounter History section and Report List interfaces, respectively. Figure S11 shows the immunization interface, which displays the user's immunization records (PH.2.5.5).

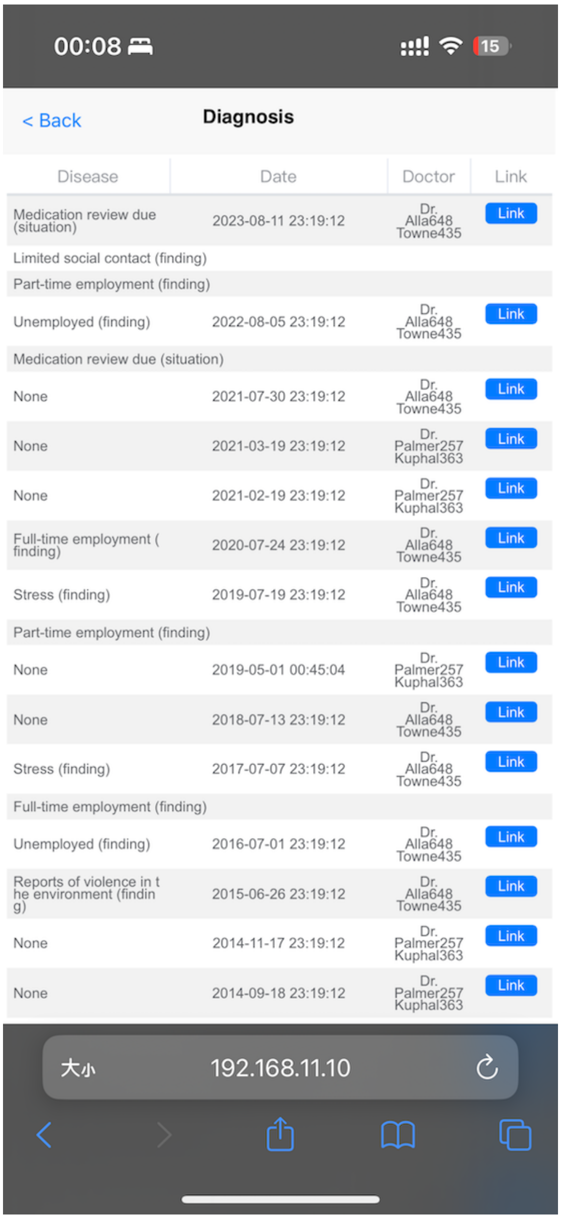

Figure S9. Diagnosis interface in the "comprehensive records" section.

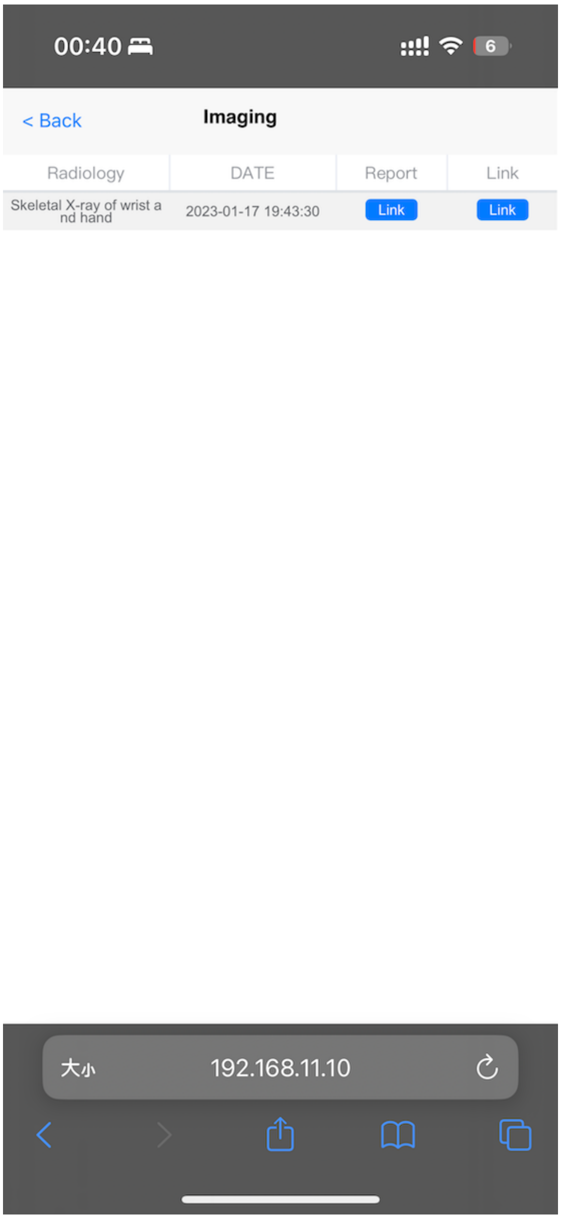

Figure S10. Imaging reports interface in the "comprehensive records" section.

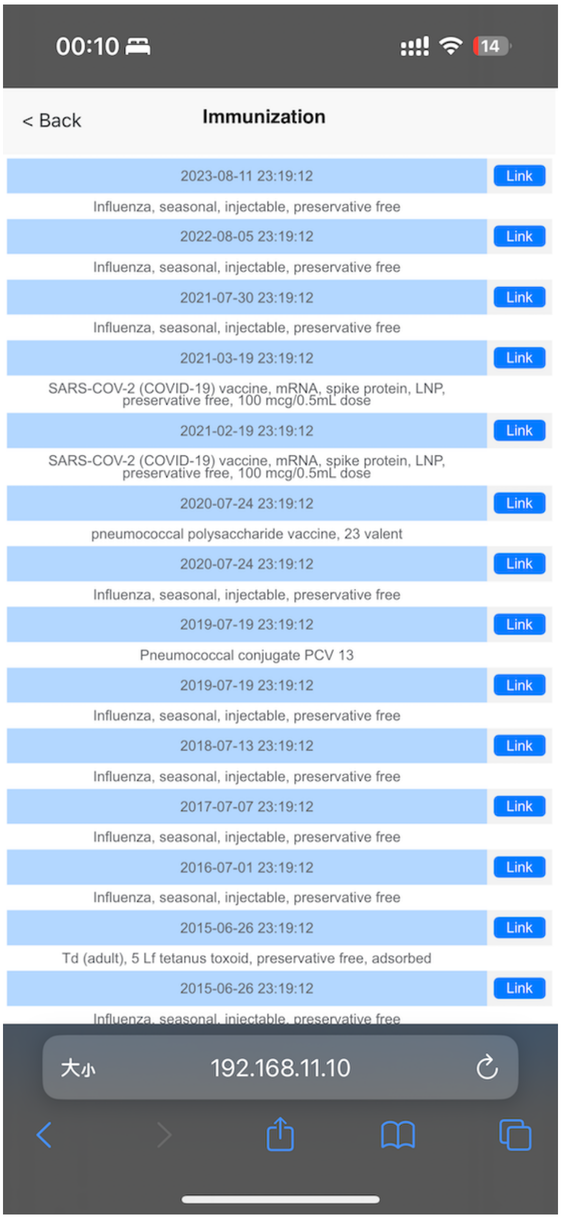

Figure S11. Immunization interface in the “comprehensive records” section.

Other Functions

In the implementation process of the PHR prototype, we referred to the requirements of standard PH.2.5.3, which calls for providing reference ranges for test results. Building on this, we introduced an innovative feature called the critical value display. Although Harahap et al. [18] have not mentioned this function, we found that standard PH.3.5.5 (notify the PHR account holder of an event or situation that may need immediate action) describes the functionality of managing health alerts. This function automatically identifies critical values in reports and prominently displays them in red in the interface. This design allows users to access abnormal reports immediately, strengthens support for proactive healthcare management, and improves user experience. As shown in Figure S12,

the PHR prototype retrieved and synchronized these critical values of test reports from the FHIR server. The Diagnostic Report resource conveys the overall outcomes of laboratory tests, whereas the Observation resource provides detailed test results. The Observation resource's "interpretation" attribute uses specific codes, such as "HH" for "Critical High" and "LL" for "Critical Low," with the "referenceRange" attribute defining the standard reference for results. In addition, we provided an auditable record function. Users can view the historical access records of personal data, including the access date, account name, and functions that were accessed. This function complies with the requirements of IN.4.

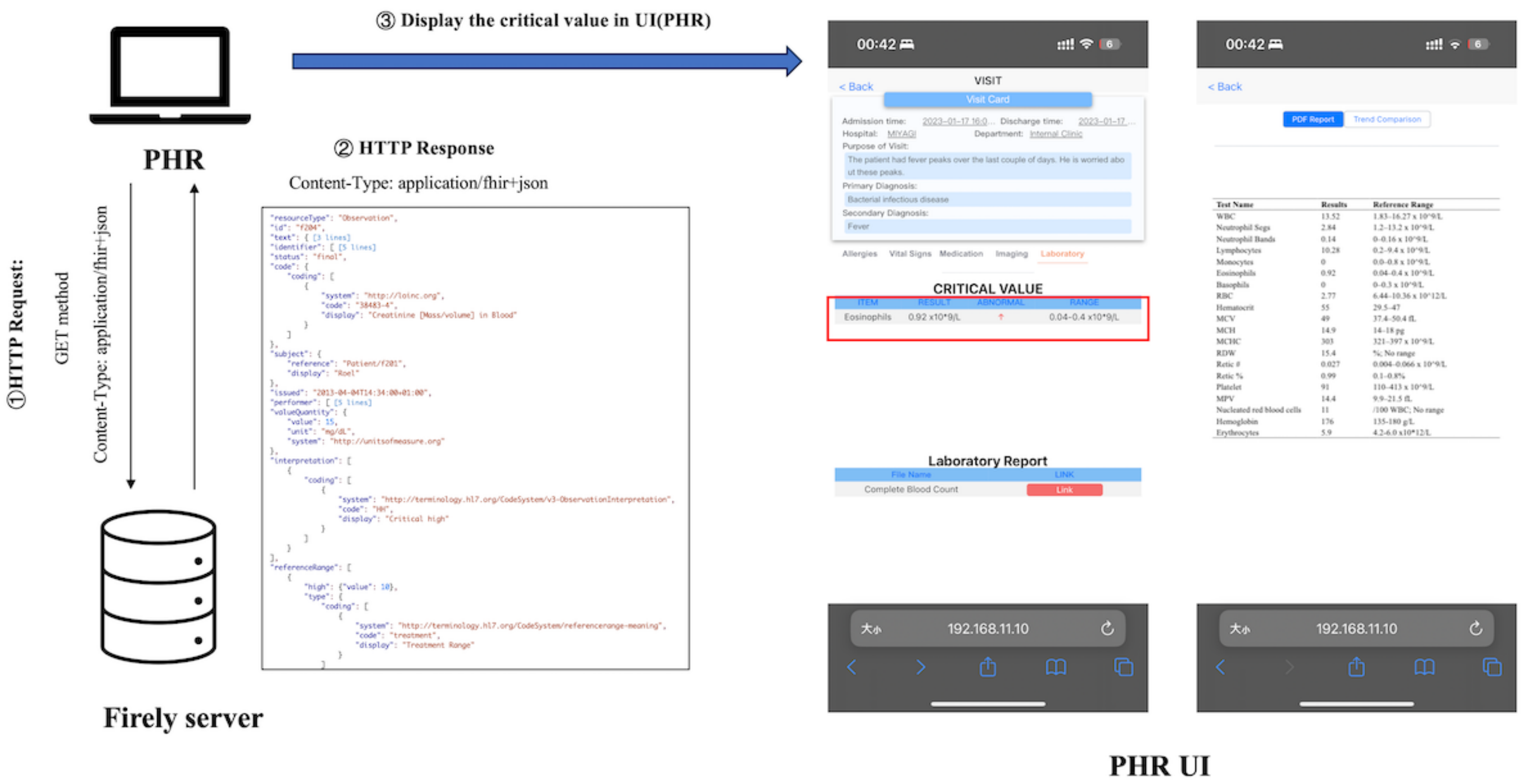

Figure S12. The PHR retrieves and displays the critical values for laboratory reports from the FHIR server.
