# Supplementary material for: Enhancing the Functionalities of Personal Health Record Systems: Empirical Study Based on the HL7 Personal Health Record System Functional Model Release 1
Source: JMIR Med Inform. 2024 Oct 9;12:e56735. doi: 10.2196/56735 (PMC11481820; doi:10.2196/56735)
Supplement: Multimedia Appendix 5 [file medinform-v12-e56735-s005.pdf]

Verification of PHR Display for Synthea Immunization Data

Figure S13 shows the method used to verify that the PHR can correctly display the Altenwerth646's immunization data from Synthea. On the left, it indicates that Altenwerth646 has seven immunization data entries in Synthea, and the right shows that the PHR can display all seven immunization data entries correctly.

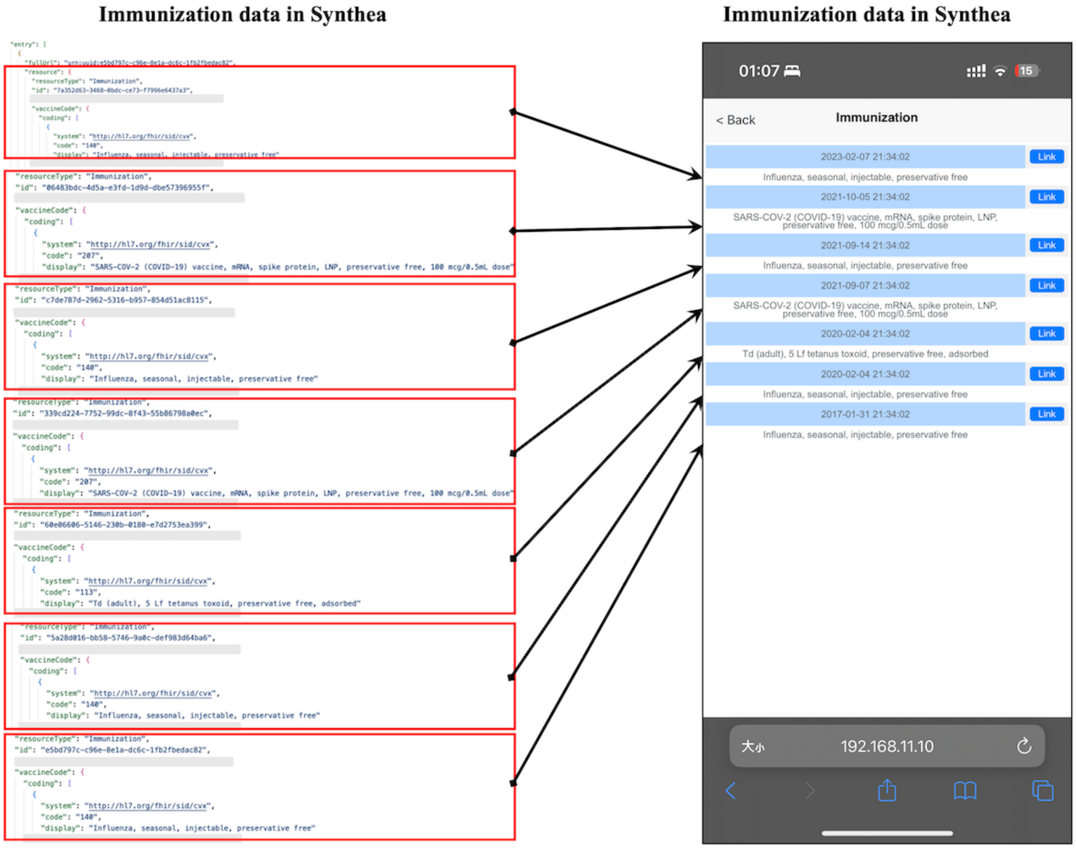

Figure S13. Verification of PHR display for Synthea Immunization data.
