# Supplementary material for: Enhancing the Functionalities of Personal Health Record Systems: Empirical Study Based on the HL7 Personal Health Record System Functional Model Release 1
Source: JMIR Med Inform. 2024 Oct 9;12:e56735. doi: 10.2196/56735 (PMC11481820; doi:10.2196/56735)
Supplement: Multimedia Appendix 6 [file medinform-v12-e56735-s006.pdf]

Multimedia Appendix 6

Table S3. Comprehensive data display coverage of PHR prototype across five patient profiles.

PHR-S FM ID#	PHR Functions		FHIR Resources	Emard19			Bradtke547			Altenwerth646			Walsh511			Parisian75		
				Data in Synthea	PHR displayed	Coverage Rate	Data in Synthea	PHR displayed	Coverage Rate	Data in Synthea	PHR displayed	Coverage Rate	Data in Synthea	PHR displayed	Coverage Rate	Data in Synthea	PHR displayed	Coverage Rate
PH.1.2	User Demographics		Patient	1	1	100%	1	1	100%	1	1	100%	1	1	100%	1	1	100%
PH.2.5.1	Diagnosis Information		Condition	11	11	100%	10	10	100%	25	25	100%	15	15	100%	24	24	100%
PH.2.5.2	Medications		MedicationRequest	7	7	100%	2	2	100%	4	4	100%	29	29	100%	13	13	100%
PH.2.5.3	Laboratory Test Reports		DiagnosticReport	2	2	100%	2	2	100%	10	10	100%	2	2	100%	15	15	100%
PH.2.5.3			Observation	22	22	100%	22	22	100%	92	92	100%	37	37	100%	124	124	100%
PH.2.5.3	Imaging Test Reports		DiagnosticReport	0	0	N/A	0	0	N/A	0	0	N/A	0	0	N/A	0	0	N/A
PH.2.5.3			Observation	0	0	N/A	0	0	N/A	0	0	N/A	0	0	N/A	0	0	N/A
PH.2.5.4	Allergy Information		AllergyIntolerance	0	0	N/A	0	0	N/A	0	0	N/A	7	7	100%	0	0	N/A
PH.2.5.5	Immunization		Immunization	17	17	100%	17	17	100%	7	7	100%	33	33	100%	15	15	100%
PH.2.5.6	Visiting Records		Encounter	17	17	100%	14	14	100%	22	22	100%	31	31	100%	26	26	100%
PH.2.5.6	Visiting Records		Practitioner	2	2	100%	3	3	100%	4	4	100%	4	4	100%	2	2	100%
PH.2.5.6	Visiting Records		Organization	2	2	100%	3	3	100%	4	4	100%	4	4	100%	2	2	100%
PH.3.1.1	Vital Signs	Body Weight	Observation	11	11	100%	10	10	100%	4	4	100%	17	17	100%	10	10	100%
PH.3.1.1		Body Height	Observation	11	11	100%	10	10	100%	4	4	100%	17	17	100%	10	10	100%
PH.3.1.1		Body Temperature	Observation	2	2	100%	0	0	N/A	1	1	100%	1	1	100%	0	0	N/A
PH.3.1.1		Body Mass Index	Observation	11	11	100%	10	10	100%	4	4	100%	8	8	100%	10	10	100%

PH.3.1.1	Blood Pressure	Observation	11	11	100%	10	10	100%	4	4	100%	17	17	100%	13	13	100%
PH.3.1.1	Heart Rate	Observation	11	11	100%	10	10	100%	4	4	100%	17	17	100%	10	10	100%
PH.3.1.1	Respiratory Rate	Observation	11	11	100%	10	10	100%	4	4	100%	17	17	100%	10	10	100%
PH.3.1.1	Pain Severity	Observation	11	11	100%	10	10	100%	4	4	100%	17	17	100%	10	10	100%
PH.3.1.1	Pediatric Head Occipital Frontal Circumference Percentile	Observation	4	4	100%	0	0	N/A	0	0	N/A	15	15	100%	0	0	N/A
PH.3.1.1	Body Mass Index (BMI) for Age	Observation	11	11	100%	10	10	100%	0	0	N/A	8	8	100%	0	0	N/A
PH.3.1.1	Pediatric Weight for Height	Observation	4	4	100%	0	0	N/A	0	0	N/A	15	15	100%	0	0	N/A
PH.3.1.1	Head Occipital-Frontal Circumference	Observation	4	4	100%	0	0	N/A	0	0	N/A	15	15	100%	0	0	N/A
		**Count:**	**183**	**183**	**100%**	**154**	**154**	**100%**	**198**	**198**	**100%**	**327**	**327**	**100%**	**295**	**295**	**100%**

Synthea Data Count : **1175**

Prototype Display Count : **1175**

Coverage Rate : **100%**
